# Proceedings of the American Medical Association, at the Third Annual Meeting, Held at Cincinnati, May, 1850

**Published:** 1850-08

**Authors:** 


### PROCEEDINGS

OF THE

### AMERICAN MEDICAL ASSOCIATION,

AT THE THIRD ANNUAL MEETING, HELD AT CINCINNATI, MAY, 1850.

---

The Association met in the "College Hall," May 7th, at 10½, A. M.

The President, Dr. Warren, in the Chair.

Dr. Strader, on behalf of the Committee of Arrangements, read a list of the delegates who had registered their names. About three hundred delegates were present.

The President delivered an Address.

On motion of Dr. Watson, of N. Y., the Constitution was read.

The rules being suspended, Dr. Watson moved that Drs. Drake, Rives, Lawson, Dodge, Strader and Richards, members of the Committee of arrangements appointed in 1849, but not belonging to the Association, be elected permanent members.

On motion of Dr. Stillé, the name of Dr. C. C. Caldwell, of Louisville, was added to the number, and the whole elected by an unanimous vote.

An invitation was presented from the "Mercantile Library," offering to the association the use of its reading room, for which, and for all similar invitations, the thanks of the Association were, on motion of Dr. Phelps, directed to be tendered.

On motion of Dr. Phelps, it was *Resolved*, That the Afternoon Session of the day commence at 4, P. M.

The names of delegates first recorded on the list of the several States were then read, on motion of Dr. Watson, and the gentlemen requested to call their colleagues together, for the purpose of constituting a Nominating Committee.

Adjourned.

*Afternoon Session.* The President in the Chair.—The Committee of Arrangements reported the names of persons recommended by various delegates as members by invitation.

Dr. White, of N. Y., moved that the subject be referred to a Special Committee of Five, who should report, at the Morning Session, the names of all who ought to be elected by the Association; which was agreed to, and the following committee appointed:

Drs. Ware, of Mass., Johnson, of Miss., Dowler of La., Parrish, of Pa., Flint, of N. Y.

The Committe of Arrangements presented an invitation to the Association from the Western Art Union to visit their rooms.

The names of delegates arrived since the morning report were read by the Committee.

The Secretary read a letter, addressed by the Secretary of the Smithsonian Institute to the President of the Association, relative to the registration of diseases, &c., throughout the United States, and offering, in behalf of the Smithsonian Institute, a room in its building as a place of meeting for the Association.

On motion of Dr. Phelps, of N. Y., that portion of the letter having reference to the Annual Meeting, was referred to the Nominating Committee.

On motion of Dr. Knight, of Conn., that part bearing upon registration was referred to the Committee on Hygiene.

The Secretary asked and obtained leave to present the Reports of the Publishing Committee and of the Treasurer.

They were accepted, and referred to the Committee on Publication, and the Resolutions appended to the Report of the Committee on Publication were adopted, as follows:

*Resolved*, That the assessment for the present year be *three dollars*.

*Resolved*, That those delegates who pay the assessment shall be entitled to one copy of the Transactions of the present year; and that the payment of two dollars, in addition, shall entitle them to two additional copies.

*Resolved*, That permanent members shall be entitled to one copy of the Transactions of the present year, on the payment of two dollars, and three copies on the payment of five dollars.

*Resolved*, That Societies which are represented at this meeting shall be entitled to copies for their members on the same terms that such copies are furnished to permanent members.

*Resolved*, That permanent members, unless present at the meeting as delegates, shall not be subject to any assessment.

*Resolved*, That any delegate who is in arrears for his annual assessment shall not be considered as a permanent member.

*Resolved*, That the several committees be requested to bring their reports correctly and legibly transcribed; and that they be required to hand them to the Secretaries as soon as they have been read.

On motion of Dr. Martin, of Indiana, the Committee of Arrangements were requested to procure another room for the meetings of the Association.

On motion of Dr. Parrish, of Pa., the Report of the Committee on Medical Education was made the order of the day for the next morning.

The Secretary presented and read a part of the Report on Hygiene.

Dr. Phelps moved the reference of the Report to the Committee on Publication.

Dr. Lawson, of Ohio, moved that it be laid on the table, for the further consideration of the meeting.

The amendment was negatived, and Dr. Phelps' motion was then adopted.

A communication from Dr. Fenner, of La., was received, accompanied by a portion of a work, now in the course of publication, upon the Meteorology, Medical Topography, and Diseases of the Southern States, and asking the co-operation of the Association.

The subject was laid upon the table, in consequence of the entrance of the Nominating Committee, prepared to report the names of officers of the Association.

The Committee, consisting of one from each State, reported the following as Officers of the Association:

*President.*—R. D. Mussey, M. D., Ohio.

*Vice Presidents.*—J. B. Johnson, Missouri, A. Lopez, Alabama, Daniel Brainard, Illinois, G. W. Norris, Pennsylvania:

*Secretaries.*—Alfred Stillé, Pennsylvania, H. W. De Saussure, South Carolina.

*Treasurer.*—Isaac Hays, Pennsylvania.

The Report was accepted, and Dr. Smith, of N. J., moved that the officers thus nominated be the Officers of the Association for the ensuing year.

After some discussion by Drs. Storer, of Mass., Yandell, of Ky., McNally, of Ohio, White and Watson, of N. Y.,

Dr. Holt moved the previous question, which was sustained, and

Dr. Smith's resolution was adopted.

On motion of Dr. Roberts, of Md., the Association then adjourned until 9, A. M., May 8.

*May 8th. Morning Session.*

Dr. Warren in the Chair.

The minutes of the previous meeting were read and approved.

The committee of Arrangements reported the names of delegates arrived since the previous report.

The following resolutions, offered by Dr. Bowditch, of Mass., were then unanimously adopted.

*Resolved*, That the American Medical Association has learned with deep regret of the death of Prof. Harrison, their late Vice President, and they hereby wish to express their high sense of the virtues, talents, and professional merit of their distinguished associate.

*Resolved*, That in dying, as he did, while engaged in ministering to the wants, and relieving the sufferings of his fellow citizens, this Association recognise in him a noble example of professional self-sacrifice.

*Resolved*, That the warmest sympathies of this Association are hereby most respectfully tendered to the family of their honored and deceased associate.

On motion of Dr. Stillé, it was

*Resolved*, That a properly authenticated copy of the resolutions be transmitted to the family of Dr. Harrison.

Dr. Blackburn, of Ky., moved that a committee of three be appointed by the Chair to introduce the newly elected officers, and to conduct the President elect to the Chair.

The Chair appointed Drs. Knight, of Con., Corbin, of Va., and Blackburn, of Ky.

The President elect having been introduced by the Committee, was presented to the Association by the President. Dr. Mussey then returned his thanks to the Association for the honor conferred upon him.

On motion of Dr. Corbin of Va., the late President and Vice Presidents were invited to take their seats upon the platform.

The following resolution, introduced by Dr. Kerfoot, of Pa., was unanimously adopted:

*Resolved*, That the thanks of the Association be tendered to the late officers, for their very gentlemanly, courteous, and efficient manner of conducting the business of the Association.

Dr. Stillé moved a suspension of the rules, for the purpose of hearing the report of the Committee on members by invitation.

Dr. Ware, chairman of the committee, made a report, concluding with the following resolutions:

*Resolved*, That all those gentlemen who have been nominated to the Association be admitted as members by invitation.

*Resolved*, That, at the next meeting of the Association, a committee shall be appointed, at an early period of the session, to whom shall be presented all nominations of members by invitation, who shall report such of them for admission as shall appear, according to a liberal interpretation of the Constitution, to have a claim to this privilege.

Dr. White, of N. Y., moved the adoption of the resolutions; but on motion of Dr. Rives, of Ohio, the resolutions were considered separately, and the first was adopted. The second, after much discussion, was, on motion of Dr. Palmer, of Michigan, indefinitely postponed.

Dr. Hooker, of Conn., offered the following:

*Resolved*, That the section in the constitution relating to members by invitation be repealed.

This lies over, according to rule, until the next meeting of the Association.

Dr. Evans, of Ky., also offered a resolution of the same purport.

The president announced the Report of the Committee on Education as the order of business of the day.

Dr. Watson asked a suspension of the rules, for the purpose of bringing before the Association the communication of Dr. Fenner, of N. O., which was refused.

Dr. Blatchford, of N. Y., presented the report of the Committee on Education, which he requested might be read by the Secretary, as the chairman of the Committee was absent. The Secretary read the report.

[This report is the production of the Chairman, Dr. Joseph Roby, Professor in the University of Maryland, and was not signed by either of his colleagues.

It refers in the first place to the previous discussions which have been entertained by the Association on this important question, and considers that they embrace a "somewhat intricate intermingling of facts and opinions." The facts include a statistical history of the number of schools, professors, students, and graduates, the means of instruction furnished, requirements for the degree, &c.

The opinions assert, in general terms, that the whole system of medical education in this country is defective and incomplete, from the fact that the schools are too numerous, their instructors too few, the time devoted to study too short, and the bestowal of honors too profuse, thus leading to a depreciation of the profession, and of the schools.

The report defines the position of the Association to be one of forbearance and conciliation towards the schools, its recommendations being advisory, and not binding; while it claims for the schools a general disposition to acquiesce in those recommendations, and to aid the association in its efforts to improve and advance the cause of sound medical learning.

In regard to the recommendations of the Association upon the subject of preliminary education, so far as these relate to the schools, the report states, "the Asaociation has attempted to obviate this difficulty, (viz., a want of suitable preliminary education,) by urging upon the schools certain recommendations, which as yet have not been fully complied with. Neither of the parties most interested seem willing to meet the responsibility. The student dislikes to have his self-complacency offended, the practitioner fears to hazard the good will of the student, and the school is too anxious lest the portal of some active rival may be found easier of access than its own. Hence, with the defect generally acknowledged and deplored, it does not appear that much has been done towards applying any efficient remedy."

In regard to increasing the number of instructors, and the period of instruction, another recommendation of the Association, the language of the report is equally discouraging.

"We do not learn," says the chairman of the committee, "that there has been a general adoption of these recommendations."

In regard to another recommendation of the Association, viz., that a more rigid test should be adopted by the schools, for admission into the profession, the report is still less satisfactory.

The writer contends that in this country, admission into all the liberal professions must be comparatively easy. "The nature of all our institutions supposes this. They must conciliate the public good will, upon which they are dependent for their existence and patronage, by a liberal exercise of their powers and privileges."

"This is especially the case with new institutions in new States. It may be deemed doubtful, therefore, whether any uniform plan of medical education can be adhered to, throughout the Union. The Northern and Middle States, having a dense and wealthy population accustomed to educational institutions, and able as well as willing to sustain them, are in a very different condition from that of the newer communities of the West and South West."

We have endeavored to lay before our readers, thus early, a faithful sketch of this report, from the great and general interest which the questions of which it treats has excited throughout the medical community. That the report fails to sustain the previous views and policy of the Association upon the subject of medical education, we think will be generally admitted. This sentiment seemed to pervade the minds of those who listened to its reading at Cincinnati, and the two members of the Committee, Dr.

Blatchford, of Troy, N. Y., and Dr. Roberts of Baltimore, who were present at the meeting, declined signing it. The former gentleman presented his views in a series of resolutions, re-affirming the previous recommendations of the Association, and urging upon Medical Colleges and upon the profession to forward and sustain them.]

Dr. Blatchford offered the following resolutions, prefacing them with the remerks that although a member of the Committee, he had not seen the report, until late on the preceding evening, and that he dissented altogether from the opinions it expressed.

*Whereas*, This Association has learned through its several committees, appointed from year to year to examine into the state of medical education in our country, that many of the medical colleges invested by law with the power of granting degrees, still continue a system of instruction which we cannot but regard as defective both in the time allotted to the delivery of lectures, in the attention paid to practical anatomy, in the facilities afforded for clinical instruction, and in the low standard of the requirements for a degree, therefore,

*Resolved*, That this Association reiterates its former recommendations upon these points, and would urge upon the medical colleges to continue their efforts to elevate the standard of medical education, by adopting such changes in their courses of instruction as shall satisfy the just and reasonable desires of the profession.

*Resolved*, That the thanks of the American Medical Association are due to the Faculties of the University of Pennsylvania, and of the College of Physicians and Surgeons of New York, and all other institutions which may have conformed to our recommendations, for their prompt response to the recommendations of the Association for the improvement of Medical Education.

[Upon these resolutions an animated debate ensued, occupying the chief part of two sessions, which was participated in by Drs. Parrish, Morris, and Stillé, of Philadelphia; Yandell and Blackburn, of Louisville, Ky.; Blatchford and Watson, of New York; Davis, of Chicago; Storer, of Boston, and Professor Gilman, of the College of Physicians and Surgeons of New York, and several other speakers, in favor of the resolutions; and by Professor Miller, of the Louisville Medical School; Mitchell of the Jefferson College of Philadelphia, and several other gentlemen, in opposition.

The following remarks by Dr. Powell are worthy of record:

"Dr. Powell, of Louisville, Ky., stated that he had long been sensible that the standard of Medical Education throughout the United States has been too low to comport with the true dignity of the Profession and the best interests of society, and he was persuaded that it is mainly owing to the fact, that young men are permitted and encouraged to enter upon the study of Medicine whose intellectual training and mental acquirements are so extremely defective as to render wholly nugatory the instruction which may be offered them. With many of these it must of necessity obtain, that medical problems are impenetrable mysteries, medical literature a dead language, and medical lectures an unintelligible jargon. We owe it to ourselves and to the community to correct this evil. The responsibility in this regard rests alike upon Practitioners and Professors, upon private and public Teachers, but not in equal degree. Of the former class, numerous as they are, widely separated, without organization and without concert, no practical and efficient measures of correction can reasonably be expected. With Medical Schools the case is widely different, *they may* and doubtless *will* always meet, by their representatives in "the American Medical Association" with the opportunity under its enlightened counsels to devise, and supported by all the moral weight of its authority with the power to enforce effective measures of reform. Among such measures, the one which would rank first in importance and work the speediest improvement in the character of the Profession, would be a *requisition* on the part of our Medical Schools, that candidates for Matriculation should be as closely examined as are the candidates for Graduation, and if found deficient in the right kind and due amount of Education, should be refused admittance into their halls. Dr. P. thought it could not be doubted that such an exhibition of moral courage and of sensible action on the part of our medical schools would silence the complaints in regard to the low standard of medical education; and every measure short of this, must end in disappointment."

In the course of the discussion, among several amendments introduced, was the substitute proposed by Dr. L. M. Lawson, of Cincinnati, Professor in the Medical College of Ohio.—*(Vide Minutes.)*

Much time having been spent on these several propositions, without coming to a direct vote, it was finally agreed to refer them to the Committee on Medical Education for next year. Subsequently a resolution offered by Dr. Morris as a substitute for the whole, was passed in committee, and, when reported to the Association, adopted. This resolution simply affirms anew the recommendations which had been made at previous meetings of the Association.—Ed. Exam.]

Dr. Roberts of Md., also a member of the committee, had never seen the report until the preceding evening, and did not entirely approve of it.

Dr. Stillé wished to correct a statement made in the Report, "That none of the Colleges of Pharmacy in the Atlantic cities seem to be in active operation." Dr. Stillé called attention to the fact that the Colleges of Pharmacy of New York and Philadelphia were in active operation, and had shown their activity, amongst other ways, by taking an efficient part in procuring the passage of the law to prevent the importation of spurious and adulterated drugs. Dr. Isaac Wood, of N. Y., also desired to say that the College of Pharmacy of New York was in active and efficient operation.

Dr. Parrish, of Pa., expressed himself at length in opposition to the doctrine of the Report, but moved that it should take the usual course, and be referred to the Committee on Publication.

Dr. Annan, of Ky., moved to amend by referring the Report and the Resolutions of Dr. Blatchford to a Select Committee, of which Dr. Parrish should be Chairman.

After much discussion, Dr. Stillé offered the following as an amendment, which was adopted:

*Resolved*, That the Report of the Chairman of the Committee on Medical Education be re-committed for correction as to matters of fact, and then handed to the Committee of Publication.

*Resolved*, That the resolutions of Dr. Blatchford be made the special order for the meeting of this afternoon.

On motion of Dr. Knight, of Ct., it was

*Resolved*, That the Committee appointed to nominate the Officers of the Association be continued, and that they be directed to nominate the several Standing Committees of the Association for the ensuing year, and also to designate the place of the next meeting of the Association.

Dr. Reyburn, of Mo., on behalf of the Medical Society of the State of Missouri, tendered an invitation from said Society to the National Medical Association to meet in St. Louis after the next annual meeting.

The Chairman of the Committee of Arrangements informed the Association that they were unable to obtain the permanent use of the only other Hall suitable for its meetings. On motion of Dr. Roberts, of Md., the Association continued to meet in the present Hall.

Adjourned to 3½ P. M.

*Afternoon Session.*

Dr. Johnson, Vice President, in the Chair.

The discussion of Dr. Blatchford's resolutions was resumed, and Dr. Miller, of Ky., moved to amend the first by inserting after the word "efforts," "and the lay members of the profession who take office students to begin their efforts," which was accepted by Dr. Blatchford.

Before coming to a vote, the Association adjourned to 9 A. M., of Thursday.

*May 9th.—Morning Session.*

Dr. Johnson, Vice President, in the Chair.

The minutes of the previous meeting were read and approved.

The Chairman of the Committee of Arrangements offered the following:

*Resolved*, That no member shall speak at one time longer than fifteen minutes, nor on any motion more than twice, without permission of the Association, which was adopted, after having been amended by changing the word "fifteen" to "ten."

The Secretary read a letter from the Dean of Cleveland Medical College, regretting the inability of their delegates to attend the meeting of the Association.

Letters of invitation were received from the Steward of the Commercial Hospital of Ohio, and Prof. C. M. Mitchell, of the Observatory, to the members of the Association, to visit their respective institutions. On motion of Dr. Eve, of Georgia, it was

*Resolved*, That 2½ o'clock, P. M., be the hour at which the Association will attend at the Observatory; and on motion of Dr. McPheeters, That in order to give the members time to visit the Observatory, when the Association adjourns, it does not meet until 4 P. M.

The President annunced the resolutions of Dr. Blatchford, amended by Dr. Miller, of Kentucky, as the first business in order.

Dr. Eve, of Ga., moved that the resolutions be indefinitely postponed, which was not adopted.

After much discussion, the previous question was moved by Dr. Edwards, and carried.

A motion for a reconsideration having been made, was carried, and the resolutions being again open for discussion, it was moved by Dr. J. R. Wood, of New York, that the Association go into Committee of the Whole, with Dr. Knight, of Ct., in the Chair. This resolution being adopted, Dr. Knight took the chair.

When the Committee rose to report, on motion of Dr. Lopez, the rules were suspended, in order to enable him to make an explanation and read a protest on behalf of the delegates of the State of Alabama, against certain statements made in the Report of the Committee on Education in 1849, and published in the volume of Transactions of that year; the protest concluding with the following resolution:

*Whereas*, The third section of the Report on Medical Education, entitled "Legal requirements exacted of medical practitioners in the several States of the Union," being discordant with the laws of the State of Alabama, now existing and in force from 1823, unrepealed; and now especially at variance with a strict sense of justice and respect to the medical faculty of that State in their professional relations and public standing,

*Resolved*, That the foregoing protest be entered upon the minutes of this present Convention, and entered on its published proceedings.

On motion of Dr. Cox, the protest was accepted, and referred to the Committee of Publication.

Dr. Lopez, second Vice President, then took the Chair, and the Chairman of the Committee of the Whole reported that they had had under consideration the preamble and resolutions of Dr. Blatchford, and certain other resolutions herewith submitted, proposed by Drs. Lawson and Drake, of Ohio, Theobald, of Md., and Gross, of Ky., which were recommended by resolution of Dr. Flint, of N. Y., to be referred to the Standing Committee for 1851; and that they afterwards adopted the accompanying resolution of Dr. Morris, of Pa., offered as a substitute for the above.

On motion, the report of the committee was adopted.

Amendment offered by Dr. Lawson, of Ohio:

*Resolved*, That this Association earnestly recommends to the members of the medical profession throughout the United States, to satisfy themselves, either by personal inquiry or the written certificate of competent persons, before receiving young men into offices as students, that they are of good moral character, and that they have acquired a good English education.

*Resolved*, That all medical colleges be advised to require of their students to exhibit evidence of a good English education prior to graduation.

*Resolved*, That medical colleges be advised to extend their lecture term to at least five months.

*Resolved*, That medical colleges be most earnestly requested to elevate the standard for graduation, and that no candidate be permitted to receive a degree who does not evince a thorough knowledge of the elements of medical science.

*Resolved*, That the schools which fail to comply with these resolutions, be refused a representative in this Association.

Amendment offered by Dr. Drake:

*Resolved*, That the medical schools of the United States should require pupils to remain till the end of the session, whatever may be its length, except when permission may be given to depart.

Amendment offered by Dr. Theobald, of Maryland:

*Resolved*, That those medical schools in the United States which have laws requiring a student to be 21 years of age, and to study medicine three years, before he is eligible to the degree of M. D., be requested to enforce said laws; and that those which have no such laws, enact them.

Amendment offered by Dr. Gross, of Kentucky:

*Resolved*, That the resolution be so far amended as to strike out the words, "*of the University of Pennsylvania*, and the *College of Physicians and Surgeons of New York*."

Resolution offered by Dr. Morris, of Pa., as a substitute, passed in Committee of the Whole, reported to the Association and adopted by it:

*Resolved*, That the recommendation of this Association at its former meetings in regard to medical education, be affirmed, and that private preceptors be still urged to receive into their offices only those duly qualified by previous education to engage in the study of medicine.

On motion of Dr. Flint, the Report of the Committee on Practical Medicine was made the special order of business for the afternoon session.

Adjourned to meet at 4 P. M.

*Afternoon Session.*

Dr. Lopez, Vice President in the Chair.

The Association met at 4 P. M.

On motion of Dr. Stillé, the Report of the Standing Committee on Surgery was made the order of the day for Friday, at 9 A. M., and certain resolutions proposed by Dr. Caldwell, the next succeeding business.

Dr. Drake announced that a case of samples of Tilden & Co.'s inspissated extracts had been presented to the Association, and that they were ready to be distributed amongst the members.

Dr. Morris, of Philadelphia, asked leave to correct an important clerical error in the resolution offered by him at the morning session in Committee of the Whole, and subsequently adopted by the Association, and that where the word *preliminary* occurs therein, the word *medical* be substituted for it. Leave was granted.

Dr. Flint, of N. Y., offered the following resolution, which was lost.

*Resolved*, That a popular address, on some medical subject, shall be annually delivered during the session of this Association, before the citizens of the place in which it shall meet, and that the Nominating Committee shall nominate some member of the Association for this purpose, with an alternate in case of his failure.

Dr. Watson, of N. Y., presented the following resolution, which was adopted.

*Resolved*, That Dr. Fenner's projected annual publication on the Diseases and Statistics of the Southern portion of the United States, meets with the cordial approbation of the American Medical Association, and is worthy of the active support and co-operation of the profession.

Dr. J. K. Mitchell, of Philadelphia, presented and read the report of the Standing Committee on Practical Medicine, which was on motion received and referred to the Committee on Publication.

The following list of nominations was presented by the Nominating Committee:

*Medical Sciences.*

Dr. Bennet Dowler, of New Orleans, Chairman.

| | |
|---|---|
| Dr. Fenner, N. O. | Dr. F. G. Smith, Philada. |
| " Upshur, Petersburg, Va. | " Carr, Canandaigua, N. Y. |
| " Johnson, Marion, Ala. | " Meers, Indianapolis, Ind. |

*Practical Medicine.*

Dr. Austin Flint, Buffalo, N. Y., Chairman.

| | |
|---|---|
| Dr. Conger, Buffalo, N. Y. | Dr. G. L. Corbin, York co., Va. |
| " R. H. Davis, Baltimore, Md. | " J. McNaughton, Albany, N. Y. |
| " W. A. Norwood, Hillsboro', N. C. | " R. Haymond, Brookville, Ind. |

*Surgery.*

Dr. Paul F. Eve, Augusta, Ga., Chairman.

| | |
|---|---|
| Dr. J. N. Simmons, Ga. | Dr. S. D. Gross, Louisville, Ky |
| " John Watson, N. Y. | " C. A. Pope, St. Louis, Mo. |
| " A. B. Palmer, Tecumseh, Mich. | " H. H. McGuire, Va. |

*Obstetrics.*

Dr. D. H. Storer, Boston, Chairman.

| | |
|---|---|
| Dr. Reynolds, Boston. | Dr. S. Thompson, Albion, Ill. |
| " H. Miller, Louisville, Ky. | " Parker, Kenoska, Wis.'n. |
| " A. J. Mullen, Napoleon, Ind. | " T. M. K. Smith, Delaware. |

*Medical Education.*

Dr. Worthington Hooker, Norwich, Ct., Chairman.

| | |
|---|---|
| Dr. T. W. Blatchford, Troy, N. Y. | Dr. J. R. Wood, N. Y. |
| | " N. S. Davis, Chicago, Ill. |
| " J. B. S. Jackson, Boston. | " C. J. Blackburn, Covington, Ky. |
| " E. W. Theobald, Baltimore. | |

*Medical Literature.*

Dr. Thomas Reyburn, St. Louis, Chairman.

Dr. W. M. McPheeters, St. Louis.

" L.M.Lawson, Cincinnati.

" N.L.Thomas, Clarksville, Tenn.

Dr. J. Couper, Newcastle, Del.

" G. Tyler, Washington, D. C.

" S. Annan, Lexington, Ky.

*Committee on Publication.*

Dr. Isaac Hays, Philada., Chairman.

Dr. Alfred Stillé, Philada.

" H. W. De Saussure, Charleston.

" N. Sanborn, Henniker, N. Hampshire.

Dr. J. R. W. Dunbar, Baltimore.

" Isaac Parrish, Philada.

" D. F. Condie, Philada.

*Committee of Arrangements.*

Dr. H. R. Frost, Charleston, Chairman.

Dr. P.C. Gaillard, Charleston.

" H. W. De Saussure, "

" W. T. Wragg, "

Dr. J. P. Jervey, Charleston.

" R. Lebby, "

" D. J. Cain, "

The Committee also recommended that the next meeting of the Association be held at Charleston, S. C.

It was moved by Dr. Bowditch, that the whole report of the committee on nominations be received and adopted.

Dr. Lawson moved that the report lie on the table, but this motion was negatived, and the original motion decided in the affirmative.

Dr. Evans, of Chicago, presented a brief report from Dr. Prioleau, Chairman of the Committee on Obstetrics, which was read and referred to the Committee on Publication, to be published or not, at their discretion.

Dr. Evans also presented a paper relating to a new instrument invented by him, called the *Obstetrical Extractor*, and which he exhibited to the Association, describing upon the manikin, the mode of manipulating it. The paper was referred to the same committee, and with like conditions as the last.

Dr. Drake, as Chairman of the Committee of Arrangements, introduced a paper by Dr. N. S. Davis, upon the question, "Has the cerebellum any special connection with the sexual propensity or function of generation?" It was read by its author, and referred to the Committee on Publication.

Adjourned till Friday at 9 A. M.

*May 10th—Morning Session.*

Dr. Johnson, Vice President, in the Chair.

The minutes of the previous meeting were read and approved.

Dr. Parsons, of R. I., Chairman of the Committee on Medical Sciences, presented the report of the committee, which was received and referred to the Committee on Publication without being read.

The Chair announced the Report of the Committee on Surgery as the special order of business.

Dr. Breckenridge, of Ohio, moved a suspension of the rules, which was lost.

Dr. Blatchford, of N. Y., asked a suspension of the rules, to enable him to present a resolution, which was refused.

Dr. Mussey, Chairman of the Committee on Surgery, stated that he had been requested by Dr. Huston, Chairman of the Committee on Spurious and Adulterated Drugs, to be permitted to read that report first, as he was about to leave the city. On motion of Dr. Smith, of N. J., the rules were suspended in order to allow the Report of the Committee on Spurious and Adulterated Drugs to be read first, to be immediately followed by the Report of the Committee on Surgery.

Dr. Huston read his report, concluding with the following resolutions:

*Resolved*, That the various State and local Medical Societies be requested annually to appoint Boards of Examiners, whose duty it shall be to procure specimens of drugs from the stores within their limits for examination, and report upon the same to their respective Societies at least once a year.

*Resolved*, That the respectable druggists and apothecaries throughout the United States be requested to take active measures for suppressing the fabrication and sale of inferior and adulterated drugs; and that it is respectfully suggested to them, wherever practicable, to form themselves into Societies or Colleges, for the promotion of pharmaceutical knowledge, and general improvement in their profession.

*Resolved*, That a committee be appointed, consisting of one member from each State here represented, whose duty it shall be to collect information with regard to spurious adulterated drugs, and report the same at the next meeting of the Association.

On motion of Dr. Stillé, the report was received, and referred to the Committee on Publication, and the resolutions were adopted.

Dr. Mussey, Chairman of the Committee on Surgery, presented and read the Report of the Committee, which, on motion, was received, and referred to the Committee on Publication.

Dr. C. C. Caldwell, of Ky., presented the following resolutions:

*Resolved*, That a committee of —— be appointed, to take into consideration, and report at the next meeting of the Association, how far the sciences of Phrenology and Mesmerism (or Animal Magnetism) are founded in truth, and to what extent a knowledge of them may be rendered subservient to the treatment and cure of diseases.

*Resolved*, That a committee of —— be appointed, to take into consideration the subject of Vital Organic Chemistry, and report at the next meeting of the Association, whether a branch of science justly entitled to that name exists, and if so, how far a knowledge of it can be rendered available to the welfare of man.

Dr. Stillé, of Pa., offered the following resolutions as a substitute for the above. Dr. Caldwell accepted the substitution, and they were adopted.

*Resolved*, That Dr. Caldwell be requested to prepare a report, to be presented at the next meeting, showing how far, in his judgment, the sciences of Phrenology and Mesmerism are founded in truth, and to what extent a knowledge of them may be rendered subservient to the treatment and cure of diseases.

*Resolved*, That Dr. Caldwell be requested to take into consideration the subject of Vital Organic Chemistry, and report to the next meeting whether, in his judgment, it can be justly called a branch of science, and if so, how far a knowledge of it can be rendered available to the welfare of man.

The Committee on Nominations reported the following names as composing the Committees:

*Committee on Indigenous Medical Botany and Materia Medica.*

Dr. A. Clapp, New Albany, Indiana, Chairman.

Dr. J. M. Bigelow, Lancaster, Ohio,

" G. Engelman, Mo.

" U. Parsons, Providence, R. I.

Dr. J. Carson, Philadelphia,

" N. B. Ives, New Haven, Conn.

" H. R. Frost, S. C.

*Committee on Hygiene.*

Dr. Jas. Moultrie, Charleston, S. C., Chairman.

Dr. P. C. Gaillard, S. C.

" D. Drake, Cincinnati, Ohio.

" J. Parrish, Burlington, N. J.

Dr. L. H. Anderson, Sumpterville, Ala.

" G. Emerson, Philada.

" H. W. De Saussure, S. C.

On motion the Report was accepted, and the nominations confirmed.

On motion, the Report of the Committee on Medical Literature was made the special order for the Afternoon Session.

On motion of Dr. Yardley, it was

*Resolved*, That the Committee on Hygiene be requested to report the best plan of warming and ventilating public and private buildings.

Dr. Blatchford, of N. Y., offered the following:

*Resolved*, That a Special Committee on Pharmacy and the Adulteration of Drugs shall be appointed by the President, consisting of seven members, of whom Dr. T. O. Edwards, of Ohio, shall be Chairman, to report at our next annual meeting; and that the Special Committee on Forensic Medicine, appointed last year under Dr. Stevens' resolution, be reappointed, and that it be optional with Dr. Stevens to continue as Chairman, or to appoint a successor, which was adopted.

On motion of Dr. Morris, of Pa., it was

*Resolved*, That it is with great satisfaction the members of this Association have observed the establishment of drug stores in which neither patent medicines, nostrums, nor other articles by which the artful and designing impose on the ignorant and credulous are exposed for sale; and that the Association recommends to its members to exert their influence in their respective spheres of action to encourage similar efforts in other places.

Dr. Phelps, of N. Y., offered the following resolution, which was adopted:

*Whereas*, The clerical profession often, though perhaps sometimes unwarily, yield their extensive influence in the community in giving currency to quackery and quack medicines, therefore

*Resolved*, That this subject be referred to the Committee on Hygiene, to consider and report at the next annual meeting of the Association.

Dr. W. Hooker, of Ct., offered the following resolutions:

*Resolved*, That the rule in relation to nostrums and secret medicines, contained in our code of medical ethics, ought to be strictly observed by the medical profession under all circumstances.

*Resolved*, That when a physician claiming to be the inventor of a new medicine, and using the measures of the common quack in effecting its sale, manages to escape censure and punishment, and to obtain even the countenace of a portion of the profession, by revealing the composition of his medicine to such of his medical brethren as may desire it, he is guilty of a dishonorable evasion of the rule referred to, and should be so considered and treated by the whole profession.

Dr. Lawson, of Ohio, moved to amend by the addition of the following:

*Resolved*, That this Association regards it as contrary to its system of ethics for medical journals to advertise nostrums, or secret remedies, although their composition may have been made known to the editor.

The resolutions and amendment were then adopted.

Adjourned to 3½ o'clock, P. M.

*May* 10*th*, *Afternoon Session.*

Dr. Johnson, Vice President in the Chair.

Dr. Miller, of Kentucky, moved a suspension of the rules, and offered the following preamble and resolution, which were adopted:

*Whereas*, Clinical instruction in medicine and surgery is now generally acknowledged to be essential to the proper qualification of students for the practice of these branches of our profession, and, whereas, it must be admitted that clinical instruction in midwifery would be equally valuable, therefore,

*Resolved,* That the Committee on Medical Education be instructed to inquire whether any practical scheme can be devised to render instruction in midwifery more practical than it has hitherto been in the medical schools of the United States, and report at the next meeting of this Association.

The secretary presented several reports, &c., which, on motion, were made the special order immediately after the report on Medical Literature.

Dr. Stillé, Chairman, presented and read the report of the Commitee on Medical Literature, concluding with the following resolutions:

*Resolved,* That the Association regards the cultivation of Medical Literature as essential to professional improvement, and as adapted to form one of the broadest lines of distinction between physicians and all pretenders to the name.

*Resolved,* That in the opinion of this Association it is equally the duty and the interest of the profession to sustain its periodical literature, both by literary contributions and subscription.

*Resolved,* That since literary excellence is best developed by literary studies, the formation of medical reading clubs, after the plan set forth in the report, is urged especially upon physicians in places where the periodical and other medical publications of the day are not readily accessible upon other terms.

*Resolved,* That the standing committee on Medical Literature be instructed to report to the Association at its next meeting, what medical work published during the year of their service, in their judgment is the most valuable, and, with the consent of the Association, such work shall be formally proclaimed by the President.

*Resolved,* That the State and local societies are hereby recommended to offer pecuniary reward, or other distinction, for the best memoir founded upon original observation.

*Resolved,* That medical colleges are hereby recommended to distingush the best inaugural thesis by a public announcement of its subject and the name of its author, and in such other manner as they deem appropriate

*Resolved*, That the sum of *one hundred dollars* raised by voluntary contribution, be offered by this Association for the best *experimental* essay on a subject connected either with Physiology or Medical Chemistry, and that a committee of seven be appointed to carry out the objects of this resolution: said committee to receive the competing memoirs until the first day of March, 1851; the authors names to be concealed from the committee: and the name of the successful competitor alone to be announced after the publicatian of the decision.

On motion, the report was accepted, and referred to the committee of publication, and the resolutions were adopted.

The Report and memorial of the committee on an international copyright law, ordered to be prepared at the last meeting of the Association, was read and accepted, and the memorial ordered to be signed by the officers and transmitted to Congress.

The Report of the special committee, appointed to consider the measures suggested in the Report on Medical Literature, for 1849, was submitted: the following resolution appended to the report was read and adopted:

*Resolved*, That in the opinion of this Association, the only legitimate means within our reach for the encouragement and maintenance of national medical literature, is to increase the standard of preliminary and professional education required of those who would enter the medical profession; to promote the circulation among the members of the profession of the medical journals of the day; to encourage the establishment of district medical libraries, and to induce every practitioner to cultivate, with care, the fields of observation and research that are within his reach.

On motion the report was accepted and referred to the committee on publication.

Dr. Gross, of Ky., offered the following preamble and resolutions which were adopted.

*Whereas*, The interests and dignity of the medical profession of the United States, as well as a true spirit of patriotism and a love of independence demand that we should use all proper and honorable means for the establishment of a national medical literature, and, whereas, we have hitherto paid too blind and indiscriminate a deference and devotion to European authorities, and not sufficiently patronized and protected our own, therefore,

*Resolved*, That this Association earnestly and respectfully recommends to the medical profession generally, and to the various medical schools in particular, the employment of native works as text books for their pupils, instead of the productions of foreign writers.

*Resolved*, That the editing of English works by American physicians, has a tendency to repress native literary and scientific authorship, and ought therefore to be discouraged by all who have at heart the objects contemplated in this preamble.

*Resolved*, That this Association will always hail with satisfaction the reprint, in their original and unmutilated form, of any meritorious works that may emanate from the British press.

On motion of Dr. Roberts, of Md., it was

*Resolved*, That a committee of three be appointed by the chair for the purpose of preparing for the action of the Association at its next convention, all unfinished business found upon its records.

Dr. Roberts, also offered the following, which was adopted:

*Resolved*, That all proposed alterations of the constitution be, and they hereby are, laid on the table for the present.

Dr. Drake, of Ohio, offered the following as an amendment to the constitution:

*Resolved*, That the second section of the Regulations of the Association be so amended, as to require that candidates for membership, by invitation, be nominated in writing by five members: that when elected they shall enjoy all the rights of delegates, and that all permanent members shall be entitled to vote. The resolution involving an amendment to the constitution lies on the table till the next meeting.

Dr. McGuire, of Va., offered the following Preamble and Resolutions, which were unanimously adopted:

*Whereas*, In every properly organized community governed by military laws, every member of it should possess a recognized position; as no military organization can be efficient and complete without including a corps of competent surgeons; as the value of their services depends in a great measure upon the degree of respect accorded to them, the common interests of our country and of our profession demand that the legal position of medical men in the army and navy should be such as will secure them due cousideration by their military associates, independently of a contingent courtesy; and as efforts are now being made to deprive medical officers in the navy of the relative position or assimilated rank conferred by a general order of the navy department, it concerns the honor of the whole profession to assist its members in the navy to obtain and secure an assimilated rank by law. Therefore,

*Resolved*, That the American Medical Association is gratified by the legislation of Congress which has conferred military rank on medical officers of the army, as it places them on an equality with officers of the several staff departments, and thus gives them a position to which the importance and dignity of the profession they represent entitles them; and it is earnestly desired that Congress, in its present session, will extend the same privileges and immunities to medical officers in the navy.

*Resolved*, That the members of the American Medical Association will exert their influence to sustain the just pretensions of their brethren to an assimilated rank in the military organizations of the country; and they would view with feelings of deep mortification a proposition from any source to deprive the medical officers of the army of any of the privileges or powers secured to them by the act of Congress approved 11th February, 1847, a law which confers upon them a protective or conservative rank, and enables them to discharge their duties more effectually.

*Resolved*, That the members of the American Medical Association hear with regret that several naval commanders have disregarded the general orders of the navy department, which place medical officers on an equality of rights and privileges, (except military command) with other officers in the navy; and they consider such resistance of the authority of the Secretary of the Navy an assumption which cannot be sanctioned by enlightened men of the present age, and should at once be put down by public opinion and by the authority of the government.

*Resolved*, That a definite position or assimilated rank, not inferior to that possessed by the medical staff of the army, should be assigned by law to medical officers in the navy, and therefore that the attention of the Senate and House of Representatives of the United States be, and is hereby invited to the subject.

*Resolved*, That copies of these resolutions be transmitted to the Secretaries of War and of the Navy, through the chiefs of the medical department of each service and the presiding officer of the Senate and House of Representatives of the United States.

On motion of Dr. Bowditch, it was

*Resolved*, That the Committee on Medical Education, be requested to report, at the next annual meeting of this Association, whether in their opinion any plan can be devised whereby medical students may receive a more thorough education in practical chemistry, than they receive at present at any of the medical colleges in the Union.

The Secretary presented the report of the Committee on Indigenous Medical Botany; a report on the vital statistics of New Orleans, by Dr. Symonds; Biographical notices of deceased physicians, by Dr. Williams, all of which were referred to the Committee on Publication; and a catalogue of Indigenous Medical Botany which was referred to the Committee on Botany.

Dr. Flint, of N. Y., submitted the following resolution, which was adopted:

*Resolved*, That the manuscript works of the late lamented Dr. Forry be referred to the Committee on Publication, to be published in connection with the Transactions of the Association provided it be deemed advisable by the committee, and consistent with the pecuniary resources of the Association.

Dr. W. L. Sutton, of Ky., nominated by Dr. Drake a permanent member, was unanimously received.

On motion of Dr. Gross, of Ky., it was

*Resolved*, That a committee be appointed to report at the next annual meeting of this Association on the propriety of recommending to the American people the importance of establishing schools of *Veterinary Medicine and Surgery*, in which the diseases of the horse, ox, dog, and other domestic animals may be investigated, and thorough, and sufficient courses of instruction delivered to such young men as may wish to qualify themselves for the practice of the Veterinary Profession.

Dr. M. L. Kreider, of Ohio, presented a protest and resolution against the vending of spurious and adulterated drugs, from the *Fairfield County Medical Institute*, which was read by the Secretary and referred to the Special Committe of which Dr. Edwards is Chairman.

The following resolution, submitted by Dr. Mead, of Illinois, was referred to the Committee on Medical Education:

*Resolved*, That the Committee on Medical Education be instructed to enquire into the expediency of recommending to the Colleges to abolish the rule which allows four years' practice to be received as an equivalent for attendance on one course of lectures, and to require all candidates for graduation to attend two full courses; also, the expediency of adopting a uniform rate of lecture fees, varying in amount only between the Colleges of the North and those of the South.

On motion of Dr. Stillé, the President was requested to appoint the several committees called for by the resolutions adopted during the session, and not otherwise provided for.

*Committee on Pharmacy and Adulteration of Drugs, under Dr. Blatchford's Resolution.*

Dr. T. O. Edwards, Cincinnati, Chairman.

| | |
|---|---|
| Dr. T. W. Blatchford, Troy, N. Y. | Dr. E. W. Theobold, Baltimore, Md. |
| " R. M. Huston, Philadelphia, | " H. R. Frost, Charleston, S. C. |
| " H. J. Bowditch, Boston, | " J. B. Johnson, St. Louis. |

*Committee on Prize Essays, under Dr. Stillé's Resolution.*

Dr. F. G. Smith, Philadelphia, Chairman.

| | |
|---|---|
| Dr. A. Stillé, Philadelphia. | Dr. F. Bache, Philadelphia, |
| " R. Bridges, " | " L. P. Yandell, Louisville, |
| " W. L. Atlee, " | " Jas. Moultrie, S. C. |

Dr. Jennings, of Mass., offered the following resolution, which was adopted:

*Resolved*, That the thanks of this Association be tendered to the Messrs. Tilden, of New Lebanon, N. Y., for samples of their medicinal extracts, which they have presented to this Association.

Dr. Morris, of Pa., presented the following resolutions, which were seconded by Dr. Yandell, of Ky., and unanimously adopted:

*Resolved*, That the thanks of this Association be tendered to the Committee of Arrangements for the careful and judicious manner in which they have provided for its accommodations, and their constant, assiduous attention to promote the convenience of its members.

*Resolved*, That we appreciate highly the hospitality and courtesy with which we have been received by the Medical Profession of Cincinnati, and assure them of the heartfelt gratitude with which we shall reflect upon the kindness they have manifested in our reception and entertainment.

*Resolved*, That the thanks of this Association be presented to the Board of Trustees of the Cincinnati Medical College, for the kindness with which they have placed their Hall at the service of this body.

On motion, the Association adjourned *sine die.*

[The analyses of the reports on Practical Medicine, Adulterated Drugs, Surgery, and Medical Literature, have been necessarily postponed for want of room. We hope to present them in our next. To the Secretary, Dr. Stillé, our thanks are duc much for valuable assistance in preparing this report. To the profession in Cincinnati the members of the Association are under the deepest obligations for the constant and unwearying efforts displayed in the promotion of their comfort and happiness. We are sure they can never be forgotten.—Ed. Exam.]

